# Adjuvant pegylated liposomal doxorubicin versus epirubicin sequential paclitaxel in triple-negative breast cancer: comparable efficacy with a distinct safety profile

**DOI:** 10.3389/fimmu.2026.1690888

**Published:** 2026-02-04

**Authors:** Shuanglong Cai, Shaohong Yu, Xiuquan Lin, Quan Zhou, Xiaoxin Zheng, Hongdan Chen, Tao Ma, Xiaogeng Chen, Hong Sun, Yong Shi

**Affiliations:** 1 Comprehensive Breast Health Center, Department of Thyroid and Breast Surgery, The Lishui Hospital of Wenzhou Medical University, The First Affiliated Hospital of Lishui University, Lishui People’s Hospital, Lishui, Zhejiang, China; 2 Department of Orthopedic Surgery, Peking Union Medical College Hospital, Peking Union Medical College and Chinese Academy of Medical Sciences, Beijing, China; 3 Department for Chronic and Noncommunicable Disease Control and Prevention, Fujian Provincial Center for Disease Control and Prevention, Fuzhou, Fujian, China; 4 Department of Gynecology, Fuzhou University Affiliated Provincial Hospital, Shengli Clinical Medical College of Fujian Medical University, Fujian Provincial Hospital, Fuzhou, Fujian, China; 5 Department of Cardiology, Fuzhou University Affiliated Provincial Hospital, Shengli Clinical Medical College of Fujian Medical University, Fujian Provincial Hospital, Fuzhou, Fujian, China; 6 First Department of Cadre Clinic, Fuzhou University Affiliated Provincial Hospital, Shengli Clinical Medical College of Fujian Medical University, Fujian Provincial Hospital, Fuzhou, Fujian, China; 7 The Third Department of Breast Cancer, Tianjin Medical University Cancer Institute and Hospital, National Clinical Research Center for Cancer, Tianjin, China; 8 Department of Breast Surgery, Fuzhou University Affiliated Provincial Hospital, Shengli Clinical Medical College of Fujian Medical University, Fujian Provincial Hospital, Fuzhou, Fujian, China; 9 Department of Pharmacy, Fujian Provincial Hospital, Shengli Clinical Medical College of Fujian Medical University, Fuzhou University Affiliated Provincial Hospital, Fuzhou, China

**Keywords:** adjuvant chemotherapy, efficacy and safety, epirubicin, pegylated liposomal doxorubicin, triple-negative breast cancer

## Abstract

**Objective:**

Pegylated liposomal doxorubicin (PLD), an improved formulation of doxorubicin, offers potential advantages in targeting and reduced systemic toxicity compared to conventional anthracyclines like epirubicin. This study aimed to compare the efficacy and safety of PLD followed by paclitaxel versus epirubicin followed by paclitaxel as postoperative adjuvant therapy for triple-negative breast cancer (TNBC).

**Methods:**

A total of 1,036 patients with TNBC who received postoperative adjuvant chemotherapy with either PLD sequential paclitaxel or epirubicin sequential paclitaxel were enrolled. The primary endpoint was disease-free survival (DFS). Adverse events were systematically documented. Multivariate Cox regression identified prognostic factors, and a predictive nomogram was developed.

**Results:**

At median follow-up, 1-, 3-, and 5-year DFS rates were 93.39%, 84.04%, and 84.04% for the PLD group versus 93.58%, 82.38%, and 81.73% for the epirubicin group (log-rank *p* = 0.58). Postoperative N stage, stromal tumor-infiltrating lymphocyte (sTIL) expression, and Ki67 expression were independent predictors of DFS. The prognostic model achieved C-indices of 0.874 (training set) and 0.853 (validation set). The PLD regimen was associated with a significantly lower incidence of most adverse events; however, nausea, mucositis, and hand-foot syndrome were more frequent in the PLD group.

**Conclusion:**

In adjuvant therapy for TNBC, PLD sequential paclitaxel demonstrated comparable efficacy to epirubicin sequential paclitaxel. However, PLD exhibited a distinct and generally more favorable safety profile, except for specific toxicities such as hand-foot syndrome and mucositis. The developed nomogram may aid in individualized prognosis prediction.

## 1 Introduction

Triple-negative breast cancer (TNBC), characterized by the absence of estrogen receptor (ER), progesterone receptor (PR), and human epidermal growth factor receptor 2 (HER2) expression, represents a distinct and aggressive subtype of breast cancer (1, 2). This subtype disproportionately affects premenopausal women and often presents at advanced stages, with higher rates of metastasis at diagnosis (3). Due to the lack of targetable receptors, TNBC is inherently resistant to traditional endocrine therapies and anti-HER2 agents, rendering chemotherapy the cornerstone of systemic treatment for these patients (4, 5).

Anthracyclines and taxanes have long formed the backbone of breast cancer chemotherapy, including regimens for TNBC (6, 7). Epirubicin, a widely used anthracycline, has demonstrated efficacy across breast cancer stages (8). However, its clinical application is constrained by cumulative toxicities, particularly cardiotoxicity—a life-threatening complication that limits long-term use (9). In contrast, pegylated liposomal doxorubicin (PLD) represents a technological advancement, encapsulating doxorubicin within polyethylene glycol-coated liposomes (10). This formulation reduces plasma levels of free doxorubicin, thereby mitigating off-target toxicity to healthy tissues while preserving antitumor efficacy (11). With an extended half-life compared to conventional doxorubicin, PLD facilitates enhanced tumor accumulation and potentially more potent cytotoxic effects (12).

Previous research has extensively investigated anthracycline-taxane combinations in TNBC, with this regimen established as a standard neoadjuvant and adjuvant approach in major clinical guidelines (13). Despite this, the optimal choice between anthracycline formulations—specifically, PLD versus epirubicin—for postoperative adjuvant therapy in TNBC remains unresolved. Although several studies have explored PLD’s role in breast cancer treatment, evidence directly comparing its efficacy and safety against epirubicin in TNBC patients remains scarce (14, 15).

This study, therefore, aimed to directly compare the efficacy and toxicity profiles of PLD-sequenced paclitaxel versus epirubicin-sequenced paclitaxel in the postoperative adjuvant treatment of TNBC. By conducting a comprehensive analysis of a large, well-characterized patient cohort, we sought to inform evidence-based decisions regarding anthracycline selection in TNBC adjuvant chemotherapy. Our findings may ultimately guide clinicians in optimizing treatment outcomes while minimizing treatment-related toxicities for this vulnerable patient population.

## 2 Materials and methods

### 2.1 Patients and data collection​

This multicenter retrospective cohort study included 1,036 female patients with stages I–III TNBC who underwent surgery at Zhejiang Province Lishui People’s Hospital, Fuzhou University Affiliated Provincial Hospital, and Tianjin Medical University Cancer Institute & Hospital between 1 January 2015 and 31 December 2020. Patients were divided into two groups based on adjuvant chemotherapy regimen: epirubicin-sequential paclitaxel (*n* = 676) and PLD-sequential paclitaxel (*n* = 360). All cases had complete clinicopathological data according to the WHO Classification of Breast Tumors (2019 edition) (16).

As this was a retrospective study, the choice between the PLD-based and epirubicin-based regimens was at the discretion of the treating physician, introducing potential selection bias. To address this, we comprehensively collected and compared baseline clinicopathological characteristics between the two groups (**Table 1**), and these were well-balanced. Furthermore, multivariate Cox regression analysis was employed to adjust for identified prognostic factors when comparing outcomes.

**Table 1:** The clinical-pathological characteristics of patients in both the training and validation sets.

Variables	Training sets	Validation sets	X2	P
	(n=725)	(n=311)		
Adjuvant treatment			0.747	0.388
Epirubicin-based	467 (64.41)	209 (67.20)		
PLD-based	258 (35.59)	102 (32.80)		
Age at diagnosis			0.527	0.468
≤35 years	71 (9.79)	26 (8.36)		
>35 years	654 (90.21)	285 (91.64)		
Menstrual status			0.389	0.533
Premenopausal	381 (52.55)	170 (54.66)		
Postmenopausal	344 (47.45)	141 (45.34)		
Family history			0.863	0.353
No	656 (90.48)	287 (92.28)		
Yes	69 (9.52)	24 (7.72)		
Surgical approach			1.475	0.225
Radical surgery	642 (88.55)	267 (85.85)		
Breast-conserving surgery	83 (11.45)	44 (14.15)		
Pathological pattern			0.102	0.951
Invasive ductal carcinoma	648 (89.38)	280 (90.03)		
Metaplastic	22 (3.03)	9 (2.89)		
Others	55 (7.59)	22 (7.07)		
Pathological T staging			0.754	0.686
T1	364 (50.21)	147 (47.27)		
T2	348 (48.00)	158 (50.80)		
T3+T4	13 (1.79)	6 (1.93)		
Pathological N staging			1.758	0.624
N0	509 (70.21)	222 (71.38)		
N1	145 (20.00)	53 (17.04)		
N2	41 (5.66)	20 (6.43)		
N3	30 (4.14)	16 (5.14)		
Tumor grade			0.006	0.936
G1+G2	312 (43.03)	133 (42.77)		
G3	413 (56.97)	178 (57.23)		
Lymph-vascular invasion			0.175	0.676
No	584 (80.55)	247 (79.42)		
Yes	141 (19.45)	64 (20.58)		
Post-sTIL levels			1.218	0.544
Low	154 (21.24)	73 (23.47)		
Intermediate	159 (21.93)	60 (19.29)		
High	412 (56.83)	178 (57.23)		
Post-Ki67 levels			0.070	0.792
Ki67 ≤ 20	73 (10.07)	33 (10.61)		
Ki67>20	652 (89.93)	278 (89.39)	0.155	0.694
Radiation therapy status				
No	556 (76.69)	242 (77.81)		
Yes	169 (23.31)	69 (22.19)		

### 2.2 Collection of clinicopathological data

Clinical data included age, menstrual status, family history, surgical procedure, adjuvant chemotherapy agents, use of adjuvant radiotherapy, and recurrence/metastasis events. Pathological data comprised pathological pattern, tumor T stage, axillary lymph node N stage (17), tumor grade, lymph-vascular invasion, stromal tumor-infiltrating lymphocyte (sTIL) expression, estrogen receptor (ER), progesterone receptor (PR), human epidermal growth factor receptor 2 (HER2), and Ki-67 status.

### 2.3 Definition of key indicators

#### 2.3.1 Clinicopathological metrics

Stromal tumor-infiltrating lymphocytes (sTILs): Classified as low (≤10%), intermediate (10%–40%), or high (>40%) expression.

ER and PR: Positive expression was defined as ≥1% immunoreactive cells by immunohistochemistry (IHC); <1% was negative (18).

HER2: Assessed by combined IHC and fluorescence *in situ* hybridization (FISH). Positivity was defined as IHC 3+ or IHC 2+ with FISH-amplified HER2; IHC 0/1+ or IHC 2+ with non-amplified HER2 was negative (19).

Ki-67: Dichotomized as low (≤20% positive cells) or high (>20% positive cells) (20).

#### 2.3.2 Inclusion and exclusion criteria

Inclusion criteria were (1) pathologically confirmed stages I–III TNBC with negative ER, PR, and HER2 expression; (2) having undergone modified radical mastectomy or breast-conserving surgery followed by adjuvant chemotherapy; and (3) availability of complete clinicopathological and follow-up data. Exclusion criteria included (1) concurrent other malignant tumors; (2) receipt of neoadjuvant therapy preoperatively; (3) severe cardiac, hepatic, or renal dysfunction; and (4) follow-up of less than 6 months or loss to follow-up.

#### 2.3.3 Adverse events

Adverse events (including myelosuppression, nausea, vomiting, cardiotoxicity, mucositis, hand-foot syndrome, and liver function abnormalities) were retrospectively extracted from electronic medical records and laboratory results and documented as present or absent.

Cardiotoxicity was specifically defined as the occurrence of any of the following: decreased left ventricular ejection fraction (LVEF ≤ 50% or a decline of ≥10% from baseline), congestive heart failure, clinically significant arrhythmia, or other cardiac dysfunction documented during clinical evaluation. Grading of adverse event severity was not performed due to the retrospective nature of the study.

### 2.4 Follow-up and survival analysis

Follow-up was conducted via outpatient visits, telephone, and electronic medical records, ending 1 June 2024. Patient survival status, recurrence/metastasis events, and treatment-related adverse events were recorded every 3–6 months.

The primary endpoint was disease-free survival (DFS), defined as the time from surgery to the first occurrence of local recurrence, regional or distant metastasis, or death from any cause. Patients without events were censored at the last follow-up.

### 2.5 Statistical analysis

Continuous variables underwent normality testing: non-normally distributed variables were reported as median (interquartile range) with the Mann-Whitney U test for between-group comparisons; normally distributed variables were reported as mean (standard deviation) with the t-test. Categorical data were expressed as proportions and compared via chi-squared test or Fisher’s exact test.

The dataset was divided into training (70%) and validation (30%) sets. In the training set, univariate and multivariate Cox regression identified independent predictors to construct a DFS prognostic model and nomograms for 1-, 3-, and 5-year DFS rates.

The dataset was randomly split into a training set (70%) and a validation set (30%). In the training set, univariate and multivariate Cox regression analyses were performed to identify independent predictors to construct a DFS prognostic model and nomograms for 1-, 3-, and 5-year DFS rates. Model discrimination was evaluated via ROC curves, and calibration was assessed using bootstrap 1000 resampling to compare predicted versus observed outcomes. Kaplan–Meier survival analysis with the log-rank test was used to estimate DFS rates and compare outcomes between treatment groups in the training set.

## 3 Results

### 3.1 Disease-free survival rates at 1-, 3-, and 5-year time points, and Kaplan–Meier curve analysis

**Figure 1:**
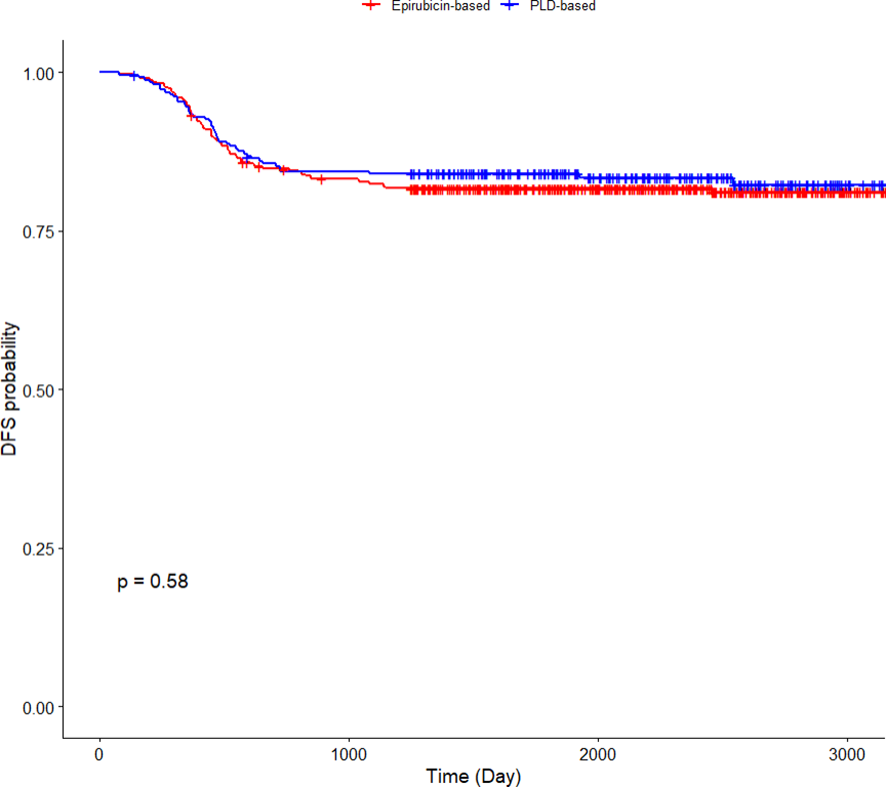
Kaplan–Meier curves for disease-free survival in patients receiving pegylated liposomal doxorubicin or epirubicin.

Among the 1,036 postoperative TNBC patients in the study cohort, the median follow-up duration was 74.4 months (95% CI: 72.3–76.2 months). During the follow-up period, DFS rates at 1, 3, and 5 years for patients receiving PLD or epirubicin are presented in **Table 2**. Kaplan–Meier curves comparing DFS between groups showed no significant difference (log-rank test, *p* = 0.58; **Figure 1**).

**Table 2:** Disease-free survival (DFS) rates of patients in the pegylated liposomal doxorubicin group and epirubicin group at different time points.

Time point	Pegylated liposomal doxorubicin group	Epirubicin group
	DFS Rate (%)	DFS Rate (%)
1 year	93.39(90.40-96.47)	93.58(91.38-95.83)
3 year	84.04(79.67-88.64)	82.38(78.99-85.92)
5 year	84.04(79.67-88.64)	81.73(78.29-85.32)

Among the 1,036 postoperative TNBC patients in the study cohort, the median follow-up duration was 74.4 months (95% CI: 72.3–76.2 months). During the follow-up period, DFS rates at 1, 3, and 5 years for patients receiving PLD or epirubicin are presented in **Table 2**. The Kaplan–Meier curves for DFS are shown in **Figure 1**. No significant difference in DFS was observed between the two groups (log-rank *p* = 0.58). The hazard ratio (HR) for disease recurrence or death in the PLD group compared to the epirubicin group was 0.902 (95% CI: 0.625–1.300).

### 3.2 Follow-up outcomes and clinicopathological characteristics in training and validation sets

The training cohort included 725 female TNBC patients with a median follow-up of 74.3 months, during which 596 DFS events and 129 deaths occurred. The validation cohort comprised 311 female TNBC patients (median follow-up: 74.4 months), with 250 DFS events and 61 deaths. Clinicopathological characteristics of patients in both sets are detailed in **Table 1**, indicating that the baseline characteristics were well-balanced between the two cohorts.

### 3.3 Nomogram model for predicting DFS in TNBC patients receiving postoperative adjuvant chemotherapy

**Figure 2:**
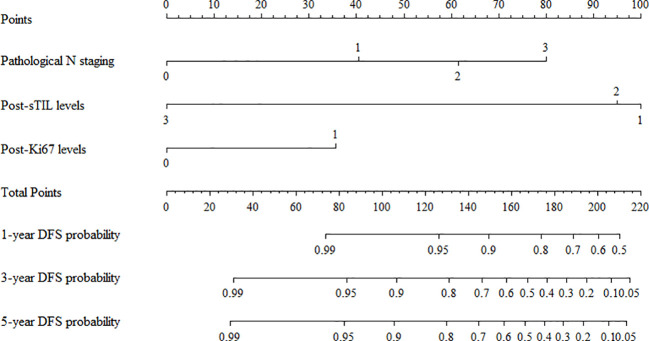
Nomogram for predicting disease-free survival in patients with triple-negative breast cancer receiving postoperative adjuvant therapy.

**Figure 3:**
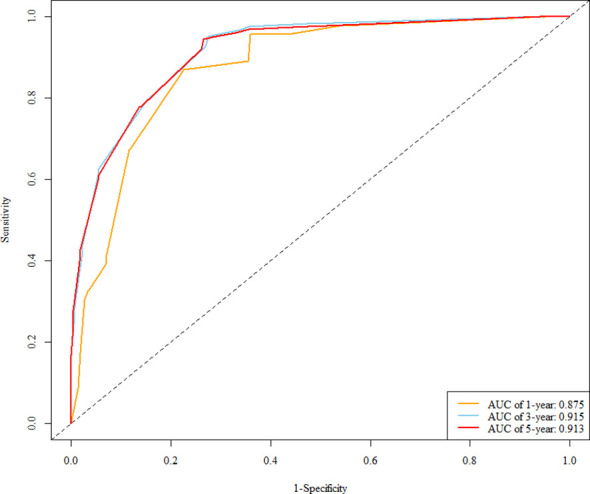
ROC curve of the training set.

**Figure 4:**
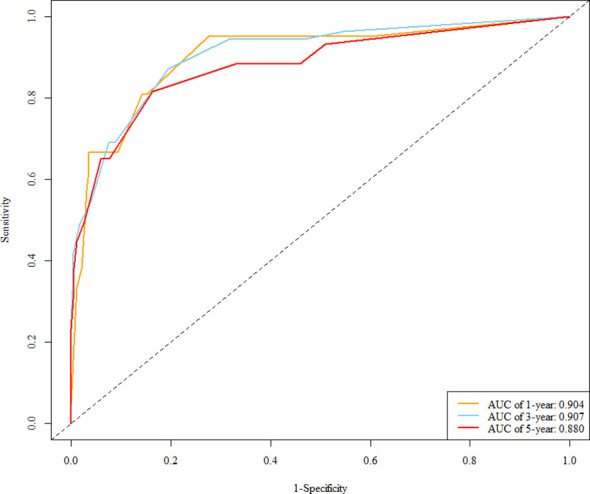
ROC curve of the validation set.

**Figure 5:**
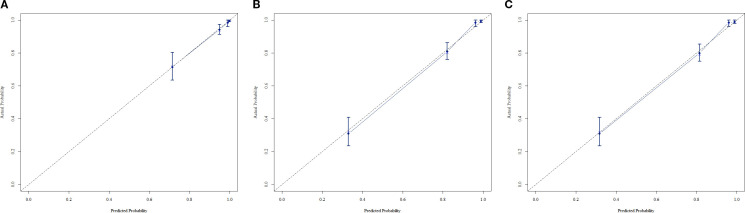
**(A)** Calibration curve of the training set at 1 year. **(B)** Calibration curve of the training set at 3 year. **(C)** Calibration curve of the training set at 5 year.

**Figure 6:**
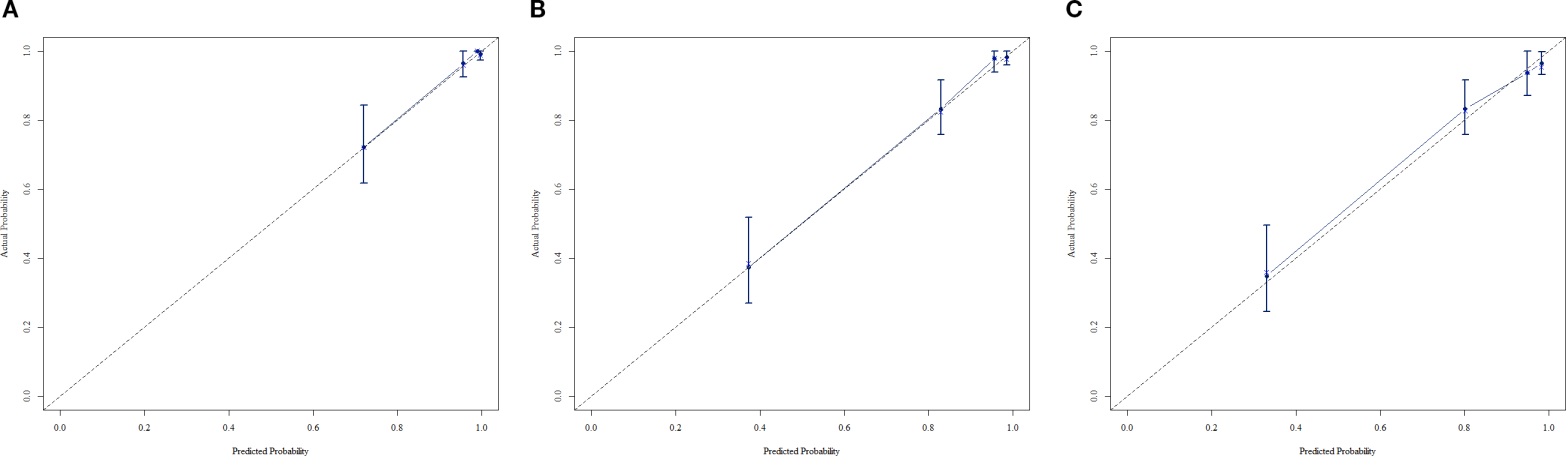
**(A)** Calibration curve of the validation set at 1 year. **(B)** Calibration curve of the validation set at 3 year. **(C)** Calibration curve of the validation set at 5 year.

In the training cohort, a multivariable Cox regression model identified postoperative N stage, stromal tumor-infiltrating lymphocyte (sTIL) expression, and Ki67 expression as independent prognostic factors (**Table 3**). Based on these variables, we developed a nomogram (**Figure 2**) to predict DFS in TNBC patients after adjuvant chemotherapy. Each subtype of these variables was assigned a point score; by inputting patient-specific values, individualized 1-, 3-, and 5-year DFS probabilities were calculated. The nomogram demonstrated good discriminative ability, with concordance indices (C-indices) of 0.874 in the training set and 0.853 in the validation set. Area under the curve (AUC) values for 1-, 3-, and 5-year survival were 0.875, 0.915, and 0.913 in the training set (**Figure 3**), and 0.904, 0.907, and 0.880 in the validation set (**Figure 4**). Calibration curves for corresponding time points are shown in **Figures 5A–C** (training set), and **Figures 6A–C** (validation set). The strong association of high Ki67 (>20%) with shortened DFS underscores the role of proliferative capacity in TNBC aggressiveness, while the protective effect of high sTILs highlights the importance of the host immune response within the tumor microenvironment.

**Table 3:** Univariate and multivariate analysis of DFS in the training set TNBC patients.

Variables	N	Events (%)	Univariate analysis		Multivariate analysis	
			HR(95%CI)	P	HR(95%CI)	P
Adjuvant treatment						
Epirubicin-based	467 (64.41)	381 (63.93)	1			
PLD-based	258 (35.59)	215 (36.07)	0.901 (0.625-1.300)	0.579		
Age at diagnosis						
≤35 years	71 (9.79)	61 (10.23)	1			
>35 years	654 (90.21)	535 (89.77)	1.288 (0.676-2.456)	0.442		
Menstrual status						
Premenopausal	381 (52.55)	317 (53.19)	1			
Postmenopausal	344 (47.45)	279 (46.81)	1.134 (0.803-1.601)	0.476		
Family history						
No	656 (90.48)	539 (90.44)	1			
Yes	69 (9.52)	57 (9.56)	0.973 (0.537-1.763)	0.929		
Surgical approach						
Radical surgery	642 (88.55)	519 (87.08)	1		1	
Breast-conserving surgery	83 (11.45)	77 (12.92)	0.353 (0.155-0.800)	0.013	0.697 (0.294-1.653)	0.412
Pathological pattern						
Invasive ductal carcinoma	648 (89.38)	112 (86.82)	1			
Metaplastic	22 (3.03)	7 (5.43)	1.988 (0.926-4.266)	0.078		
Others	55 (7.59)	10 (7.75)	1.086 (0.568-2.073)	0.803		
Pathological T staging						
T1	364 (50.21)	324 (54.36)	1		1	
T2	348 (48.00)	265 (44.46)	2.333(1.599-3.402)	<0.001	1.441 (0.972-2.138)	0.069
T3+T4	13 (1.79)	7 (1.17)	5.393(2.286-12.724)	<0.001	2.385 (0.92-6.183)	0.074
Pathological N staging						
N0	509 (70.21)	465 (78.02)	1		1	
N1	145 (20.00)	103 (17.28)	3.888 (2.547-5.934)	<0.001	3.038 (1.684-5.48)	<0.001
N2	41 (5.66)	24 (4.03)	6.007 (3.431-10.518)	<0.001	5.776 (2.713-12.298)	<0.001
N3	30 (4.14)	4 (0.67)	20.919 (12.737-34.357)	<0.001	9.101 (4.606-17.987)	<0.001
Tumor grade						
G1+G2	312 (43.03)	244 (40.94)	1		1	
G3	413 (56.97)	352 (59.06)	0.672 (0.475-0.949)	0.024	1.123 (0.781-1.615)	0.532
Lymph-vascular invasion						
No	584 (80.55)	525 (88.09)	1		1	
Yes	141 (19.45)	71 (11.91)	6.318 (4.462-8.946)	<0.001	1.109 (0.65-1.892)	0.703
Post-sTIL levels						
Low	154 (21.24)	90 (15.10)	1		1	
Intermediate	159 (21.93)	103 (17.28)	0.745 (0.520-1.067)	0.108	0.792 (0.534-1.175)	0.246
High	412 (56.83)	403 (67.62)	0.039 (0.020-0.079)	<0.001	0.043 (0.021-0.089)	<0.001
Post-Ki67 levels						
Ki67 ≤ 20	73 (10.07)	68 (11.41)	1		1	
Ki67>20	652 (89.93)	528 (88.59)	3.019 (1.235-7.381)	0.015	2.822 (1.105-7.209)	0.030
Radiation therapy status						
No	556 (76.69)	464 (77.85)	1			
Yes	169 (23.31)	132 (22.15)	1.349 (0.921-1.976)	0.124		

### 3.4 Analysis of adverse drug reactions

Based on medical records, common adverse events included myelosuppression, nausea, vomiting, cardiotoxicity, mucositis, hand-foot syndrome, and liver function abnormalities (**Table 4**). The PLD group had higher incidences of hand-foot syndrome (24.7% vs. 8.5%) and mucositis (22.5% vs. 17.4%) compared with the epirubicin group. Conversely, the PLD group exhibited lower rates of myelosuppression, vomiting, cardiotoxicity, and liver function abnormalities, although the incidence of nausea was higher (5.5% vs. 2.0%).

**Table 4:** Drug-related adverse reactions.

Adverse event	PLD-based regimen (N=360)	Epirubicin-based regimen (N=676)
Myelosuppression	18 (5%)	54 (7.9%)
Nausea	20 (5.5%)	14 (2.0%)
Vomiting	1 (0.2%)	8 (1.1%)
Cardiotoxicity	8 (2.2%)	46 (6.8%)
Mucositis	81 (22.5%)	118 (17.4%)
Hand-foot syndrome	89 (24.7%)	58 (8.5%)
Abnormal liver function	0 (0%)	9 (1.3%)

PLD, pegylated liposomal doxorubicin.

## 4 Discussion

TNBC, defined by the absence of estrogen receptor (ER), progesterone receptor (PR), and human epidermal growth factor receptor 2 (HER2) expression, represents an aggressive subtype with limited therapeutic options and poor prognosis (21, 22). Postoperative adjuvant chemotherapy remains a cornerstone of treatment; however, the efficacy and safety profiles of different regimens vary substantially. PLD sequential paclitaxel and epirubicin sequential paclitaxel are commonly used adjuvant chemotherapy regimens for TNBC. PLD, a nanoparticle-mediated antineoplastic agent, prolongs circulation time and enhances tumor accumulation via liposomal encapsulation, demonstrating higher rates of pathological complete response (pCR) in select clinical studies (23, 24). In contrast, our adjuvant study found comparable DFS between the two strategies. This divergence invites a refined discussion on the relationship between pCR and long-term outcomes in TNBC. While achieving pCR is a well-validated surrogate for survival benefit in TNBC, supported by major trials and meta-analyses (25, 26), its translation is not absolute. The observed discrepancy likely underscores the fundamental distinction between neoadjuvant and adjuvant therapy contexts. Neoadjuvant therapy evaluates tumor response *in situ* (pCR), whereas adjuvant therapy tests the ability to prevent recurrence after macroscopic tumor removal (DFS). The pharmacological properties and efficacy drivers of a drug may differ between these settings. Factors such as differences in study populations, baseline tumor burden, and the specific endpoints measured (pCR vs. DFS) contribute to the variance in results. Thus, the comparable DFS in our adjuvant study does not contradict the potential utility of PLD in neoadjuvant therapy but highlights that therapeutic efficacy must be evaluated within its specific clinical context. Further research is warranted to delineate the distinct roles and optimal applications of liposomal versus conventional anthracyclines across the continuum of TNBC management.

Regarding safety, our study revealed distinct adverse event profiles between the two regimens. The PLD group exhibited higher incidences of hand-foot syndrome (24.7% vs. 8.5%) and mucositis (22.5% vs. 17.4%), likely due to prolonged drug retention and accumulation in skin and mucosal tissues (27). A meta-analysis of multicenter data attributed these effects to the unique nanoparticle structure of PLD, which causes drug sequestration in cutaneous capillary beds (28). Conversely, the PLD group had lower rates of myelosuppression, vomiting, cardiotoxicity, and liver function abnormalities, consistent with previous findings that liposomal formulations mitigate anthracycline-induced cardiotoxicity (9, 29). Notably, nausea occurred more frequently in the PLD group (5.5% vs. 2.0%), potentially related to differential drug metabolism and gastrointestinal mucosal irritation mechanisms (30). These findings highlight the critical importance of individualized treatment selection, balancing efficacy with the specific toxicity profile most suitable for a given patient. It is important to note that this was a retrospective analysis where treatment allocation was not randomized, which may introduce selection bias. Although we observed balanced baseline characteristics between the two groups and used multivariate analysis to adjust for known prognostic factors, unmeasured confounders (such as subtle differences in performance status, physician preference based on unrecorded patient factors, or precise drug dose intensity) could still influence the outcomes. A prospective, randomized trial would be the gold standard to definitively confirm these findings.

Previous studies have identified multiple factors influencing DFS in TNBC, including tumor stage, molecular marker expression, and immune microenvironment (31–33). Our study employed Cox regression analysis to identify postoperative N1–N3 nodal stage, high stromal tumor-infiltrating lymphocyte (sTIL) expression, and Ki67 >20% as key independent predictors of DFS. These findings have significant implications for precise prognosis assessment and personalized treatment planning (21, 22).

In the TNM staging system, nodal (N) stage reflects regional lymph node involvement. Our results showed a strong association between N1–N3 stages and DFS, indicating that increased lymph node metastasis burden elevates the risk of systemic dissemination and shortens DFS (34, 35). This aligns with extensive literature demonstrating that regional lymph node metastasis is a robust prognostic marker across cancers, including TNBC (36). Longitudinal studies of breast cancer patients have consistently reported higher recurrence rates and lower 5-year survival among those with nodal involvement, underscoring the clinical importance of accurate lymph node assessment in TNBC.

Tumor-infiltrating lymphocytes (TILs) are integral to the tumor microenvironment and play a central role in anti-tumor immunity. Our study found that high postoperative sTIL expression correlated with longer DFS. Mechanistically, elevated sTILs enhance tumor cell recognition and cytotoxicity, inhibit angiogenesis, and delay recurrence (37, 38). Prior research has shown that TNBC patients with high TILs exhibit higher pCR rates and improved survival outcomes. However, the context-dependent nature of TIL function across patients and tumor stages warrants further investigation.

Ki67, a nuclear protein associated with cell proliferation, directly reflects tumor cell mitotic activity. Our analysis revealed that Ki67 >20% significantly shortened DFS, consistent with its role in promoting tumor aggressiveness and metastasis. Multiple breast cancer cohorts have reported higher recurrence rates and shorter DFS in patients with high Ki67 expression (39, 40), supporting its utility as a prognostic biomarker for treatment stratification.

## 5 Limitations

This study has several limitations. First, due to its non-randomized, retrospective design, residual confounding from unmeasured variables (e.g., detailed performance status, cardiac risk profiles, physician preference, socioeconomic factors, precise chemotherapy dose intensity, compliance, or patient-reported outcomes) may persist despite our efforts to adjust for known prognostic factors. The lack of granular data also precluded the use of propensity score matching or sensitivity analyses to further mitigate selection bias. Second, reliance on medical records introduces potential information bias and prevented the assessment of patient-reported outcomes such as quality of life, which are important for a holistic evaluation of treatment benefit. Third, the analysis was limited to selected clinicopathological factors and did not include lifestyle, genetic, or additional molecular variables that could influence DFS. Fourth, the follow-up duration was insufficient to evaluate long-term survival and late-onset toxicities. Finally, although the nomogram showed good performance in internal validation, external validation in larger, independent, prospective multicenter cohorts is necessary to confirm its generalizability and clinical utility before widespread adoption.

## 6 Conclusion

In postoperative adjuvant therapy for stage I-III TNBC, PLD sequential paclitaxel and epirubicin sequential paclitaxel showed comparable DFS outcomes, supporting the role of nanoparticle formulations in oncology. However, their distinct toxicity profiles require careful consideration: PLD offers advantages in myelosuppression, cardiotoxicity, and liver function, but increased vigilance for hand-foot syndrome and mucositis is necessary. The developed nomogram provides a practical tool for predicting individual DFS probabilities, which could aid in patient counseling and follow-up planning. Future prospective, large-scale studies with external validation are warranted to confirm these findings and further refine adjuvant therapy strategies for TNBC.

## Data Availability

The original contributions presented in the study are included in the article/supplementary material. Further inquiries can be directed to the corresponding authors.
